# 

**Published:** 1973-01

**Authors:** 


					I
K 323
NUARY 1973
"''(v' " j?fe -rl'>Vi>^
VOL. 88 (i) No. 325
?? ' . SL>. met ?
" ? ' ???" '? L' .V

				

